# Critical Current Density and Meissner Effect of Smart Meta-Superconductor MgB_2_ and Bi(Pb)SrCaCuO

**DOI:** 10.3390/ma15030972

**Published:** 2022-01-27

**Authors:** Honggang Chen, Yongbo Li, Yao Qi, Mingzhong Wang, Hongyan Zou, Xiaopeng Zhao

**Affiliations:** Smart Materials Laboratory, Department of Applied Physics, Northwestern Polytechnical University, Xi’an 710129, China; 2017100698@mail.nwpu.edu.cn (H.C.); 2014100616@mail.nwpu.edu.cn (Y.L.); qiyao@mail.nwpu.edu.cn (Y.Q.); wangmingzhongsuper@163.com (M.W.); 2019202522@mail.nwpu.edu.cn (H.Z.)

**Keywords:** smart meta-superconductor, transport critical current density, Meissner effect, critical transition temperature

## Abstract

The smart meta-superconductor MgB_2_ and Bi(Pb)SrCaCuO increase the superconducting transition temperature (*T_C_*), but the changes in the transport critical current density (*J_C_*) and Meissner effect are still unknown. Here, we investigated the *J_C_* and Meissner effect of smart meta-superconductor MgB_2_ and Bi(Pb)SrCaCuO. The use of the standard four-probe method shows that Y_2_O_3_:Eu^3+^ and Y_2_O_3_:Eu^3+^+Ag inhomogeneous phase significantly increase the *J_C_*, and *J_C_* decreases to a minimum value at a higher temperature. The Meissner effect was measured by direct current magnetization. The doping of Y_2_O_3_:Eu^3+^ and Y_2_O_3_:Eu^3+^+Ag luminescent inhomogeneous phase causes a Meissner effect of MgB_2_ and Bi(Pb)SrCaCuO at a higher temperature, while the non-luminescent dopant reduces the temperature at which samples have Meissner effect. The introduction of luminescent inhomogeneous phase in conventional MgB_2_ and copper oxide high-temperature Bi(Pb)SrCaCuO superconductor increases the *T_C_* and *J_C_*, and Meissner effect is exerted at higher temperature. Therefore, smart meta-superconductivity is suitable for conventional and copper oxide high-temperature superconductors.

## 1. Introduction

Superconductivity has greatly expanded people’s understanding of condensed matter physics and greatly promoted the progress of industrial technology [1,2]. In the superconducting state, superconductors have zero-resistance characteristic and complete diamagnetism (Meissner effect) [3,4,5,6,7]. The zero-resistance characteristic and Meissner effect are both independent and closely related to each other. A material must satisfy the zero-resistance characteristic and the Meissner effect simultaneously to determine whether it is a superconductor [2].

All superconductors transition from a superconducting state to a non-superconducting state may have their own characteristic parameters: critical transition temperature (*T_C_*), critical current density (*J_C_*), and critical magnetic field (*H_C_*) [1,3]. Critical current density *J_C_* is an important parameter to characterize superconductivity, and it is also one of the main parameters to measure the performance of superconducting materials in engineering technology applications. In scientific research, the electric transport and hysteresis loop methods are two widely used methods to measure the critical current [8]. The electric transport method is accurate and reliable, and it is used in the international critical current measurement standard. Electrical transport measurement usually uses the four-probe method. After a certain current *I* is input into the sample through the current lead, the voltage *V* of the sample is measured. The critical current *I_C_* is defined as the transport current when a significant drift voltage exists [9,10,11]. The current–voltage (*I*–*V*) curve is used to determine the critical current *I_C_*, which then enables the determination of the critical current density *J_C_* [12,13].

Since the discovery of superconductivity, increasing transition temperature and transport critical current density of the superconductor has been the main research direction of superconductivity. At present, the commonly used methods to increase the superconducting transition temperature are to modify existing superconductors and develop new superconducting materials, such as doping Al [14], C [15], and Li [16] in MgB_2_ and doping Cs [17], SnO_2_ [18], and ZrO_2_ [19] in a BiSrCaCuO superconductor. However, the dopants are unstable at a high temperature and will react with the superconductor. Thus, this method cannot increase the superconducting transition temperature. In recent years, researchers have found that hydrides have a higher transition temperature under high pressure. For example, a superconductivity of 203 K was observed in a sulfur hydride system at 155 GPa [20], superconductivity of 250 K in LaH_10_ at 170 GPa [21], and the room temperature superconductivity of 287.7 K in a carbonaceous sulfur hydride system at 267 GPa [22]. Although this approach can achieve a higher superconducting transition temperature and even room-temperature superconductivity, the extremely high pressure and small sample size limits its further applications. Thus far, there is no particularly good strategy for increasing the superconducting transition temperature. Chemical doping is the easiest way to change the *J_C_* of superconductor because it does not require costly raw materials or complex technologies. For example, doping graphene [23] and Dy_2_O_3_ [24] in MgB_2_ and doping Al_2_O_3_ [25], MgO [26], and SiC [27] in BiSrCaCuO decrease its *J_C_* in self-field. Meanwhile, doping anthracene into MgB_2_ [28] and doping Cr_2_O_3_ [29], SnO_2_ [30], and ZnO [31] in BiSrCaCuO will increase its *J_C_* in self-field. Although the *J_C_* of superconductor increases or decreases in self-field due to chemical doping, the corresponding *T_C_* decreases. Therefore, no particularly effective method can increase the *T_C_* and *J_C_* at the same time in self-field.

A metamaterial is a kind of composite material with an artificial structure. It exhibits supernormal physical properties that natural materials do not possess, and these supernormal properties are determined by special artificial structures [32,33]. Recently, Smolyaninov et al. proposed that a higher transition temperature can be obtained by constructing a metamaterial superconductor with an effective dielectric constant of nearly zero or hyperbolic metamaterial superconductor [34,35,36]. In 2007, our research group proposed to introduce an inorganic ZnO electroluminescence (EL) material in the high-temperature Bi(Pb)SrCaCuO superconductor to influence the Bi(Pb)SrCaCuO superconducting transition temperature [37,38,39]. Y_2_O_3_ is a non-electroluminescent material and can become a kind of electroluminescent material after the addition of a small amount of Eu^3+^ ions as the luminous center. Y_2_O_3_:Eu^3+^ is a rare earth luminescent material with excellent performance. In addition, the preparation of Y_2_O_3_:Eu^3+^ into Y_2_O_3_:Eu^3+^+Ag topological luminophore can further improve its EL performance. With the development of a metamaterial, we constructed a MgB_2_ and Bi(Pb)SrCaCuO smart meta-superconductor in recent years. The smart meta-superconductors are composed of superconducting particles and Y_2_O_3_:Eu^3+^ and Y_2_O_3_:Eu^3+^+Ag luminescent inhomogeneous phases. We doped Y_2_O_3_:Eu^3+^ and Y_2_O_3_:Eu^3+^+Ag EL materials in conventional MgB_2_ and high-temperature Bi(Pb)SrCaCuO superconductors [37,38,39,40,41,42,43,44]. The research results showed that the doping of Y_2_O_3_:Eu^3+^ and Y_2_O_3_:Eu^3+^+Ag EL materials increases the *T_C_* of MgB_2_ and Bi(Pb)SrCaCuO. The *T_C_* of MgB_2_ is increased by 1.2 K, and the zero resistance temperature *T_C,0_* and the onset transition temperature *T_C,on_* of Bi(Pb)SrCaCuO are increased by 4 and 6.3 K, respectively. We believe that this result is due to superconducting particles acting as microelectrodes to excite the EL of the luminescent inhomogeneous phases under the action of an external electric field. EL energy injection promotes the formation of electron pairs. Accordingly, the *T_C_* of MgB_2_ and Bi(Pb)SrCaCuO can be increased via EL [43,44].

In previous studies, the *T_C_* of MgB_2_ and Bi(Pb)SrCaCuO were increased by constructing a smart meta-superconductor. However, the *J_C_* and Meissner effect were not studied. This study investigates the *J_C_* and Meissner effect of MgB_2_ and Bi(Pb)SrCaCuO smart meta-superconductor. The results show that the addition of Y_2_O_3_:Eu^3+^ and Y_2_O_3_:Eu^3+^+Ag luminescent inhomogeneous phase increases the *T_C_* of MgB_2_ and Bi(Pb)SrCaCuO, while increasing the *J_C_* and the *J_C_* of the luminescent inhomogeneous phase doped samples decreases to a minimum value at higher temperatures. The direct current (DC) magnetization data indicate that Y_2_O_3_:Eu^3+^ and Y_2_O_3_:Eu^3+^+Ag luminescent inhomogeneous phase doping causes a Meissner effect of MgB_2_ and Bi(Pb)SrCaCuO at a higher temperature, while non-luminescent dopant doping reduces the temperature of the Meissner effect.

## 2. Experiment

### 2.1. Preparation and Characterization of Pure MgB_2_ and Doping MgB_2_ Superconducting Samples

Using MgB_2_ with three different particle sizes, three series of samples doped with a luminescent inhomogeneous phase or non-luminescent dopant were prepared by ex-situ sintering, and the samples were marked as ^a^MgB_2_ (Φ_a_ < 30 μm), ^b^MgB_2_ (Φ_b_ < 15 μm), and ^c^MgB_2_ (Φ_c_ < 5 μm) series samples, and the thickness of prepared bulk samples is 1.2 mm. X-ray diffraction (XRD) and scanning electron microscope (SEM) characterization show that the main phase of all samples is MgB_2_, a small amount of MgO impurity phase is detected, and the particle sizes of ^a^MgB_2_, ^b^MgB_2_, and ^c^MgB_2_ decrease sequentially. The curve of the temperature dependence of resistivity (*R*–*T*) shows that the non-luminescent dopants Y_2_O_3_ and Y_2_O_3_:Sm^3+^ doping decreases the *T_C_* of MgB_2_, while the luminescent inhomogeneous phase Y_2_O_3_:Eu^3+^ and Y_2_O_3_:Eu^3+^+Ag doping increases the *T_C_* of MgB_2_ in different amplitudes. The preparation process and related characterization of pure MgB_2_ and doped MgB_2_ samples were described in [44].

### 2.2. Preparation and Characterization of Pure B(P)SCCO and Doping B(P)SCCO Superconducting Samples

Three series of pure B(P)SCCO and doped B(P)SCCO superconducting samples with different particle sizes were prepared using three kinds of B(P)SCCO raw materials with successively decreasing particle sizes, and the samples were marked as A (A1–A6), B (B1–B6), C (C1–C7) series samples. The thickness of prepared bulk samples is 1.2 mm. XRD and SEM show that the main phase of the prepared samples is the high-temperature phase Bi2223, which contains a small amount of the low-temperature phase Bi2212, and the microstructure is a randomly distributed plate-like structure. The particle sizes of A, B, and C series samples decrease in turn. *R*–*T* test indicates that the non-luminescent dopants Y_2_O_3_ and Y_2_O_3_:Sm^3+^ doping decreases the *T_C_* of B(P)SCCO, while the *T_C_* of B(P)SCCO increases with the doping of luminescent inhomogeneous phases Y_2_O_3_:Eu^3+^ and Y_2_O_3_:Eu^3+^+Ag. The preparation process and related characterization of samples were described in [43,45].

### 2.3. Testing of Transport Critical Current Density and Meissner Effect

As usually conducted in superconducting systems, transport critical current density (*J_C_*) was determined by *I*–*V* measurements at different temperatures (below the onset transition temperature *T_C,on_*) with a voltage criterion of 1 μV/cm [8,12,13]. Subsequently, DC magnetization measurements were performed on the prepared samples [46]. The samples were cooled slowly in a magnetic field of 1.8 and 2.5 mT parallel to the plane, and data were collected during heating. All samples are fully diamagnetic.

## 3. Results and Discussion

**Figure 1:**
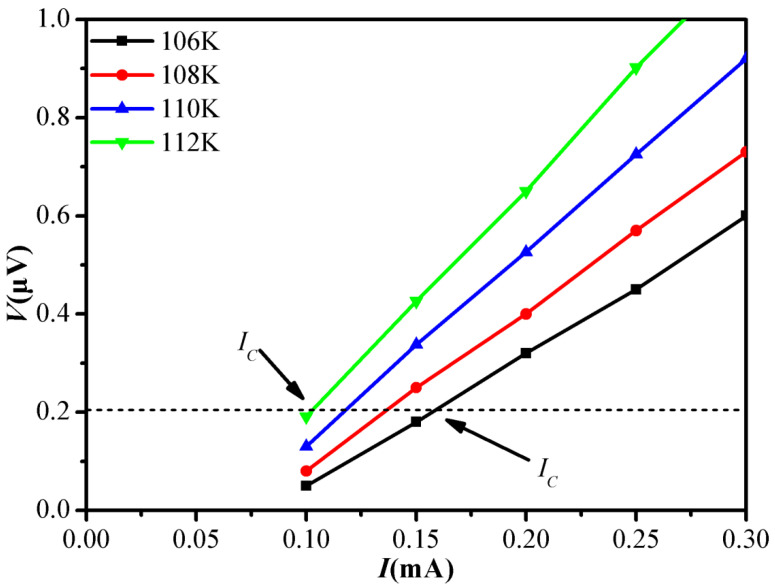
*I*–*V* curves of pure B(P)SCCO (A1) at 106, 108, 110, and 112 K.

As usually conducted in superconducting systems, *I*–*V* curves of superconductors at different temperatures were used to extract *I_C_*. The *I*–*V* curves of the samples were tested by a four-probe method. A test current was applied to the prepared samples, and a Keithley digital nanovoltmeter was used to measure the high resolution voltage. Figure 1 shows the *I*–*V* curves of pure B(P)SCCO (A1) at different temperatures (106, 108, 110, and 112 K). The extraction criteria of *I_C_* are given in this figure, and the *I_C_* of all samples prepared is obtained using this criterion in this experiment.

**Figure 2:**
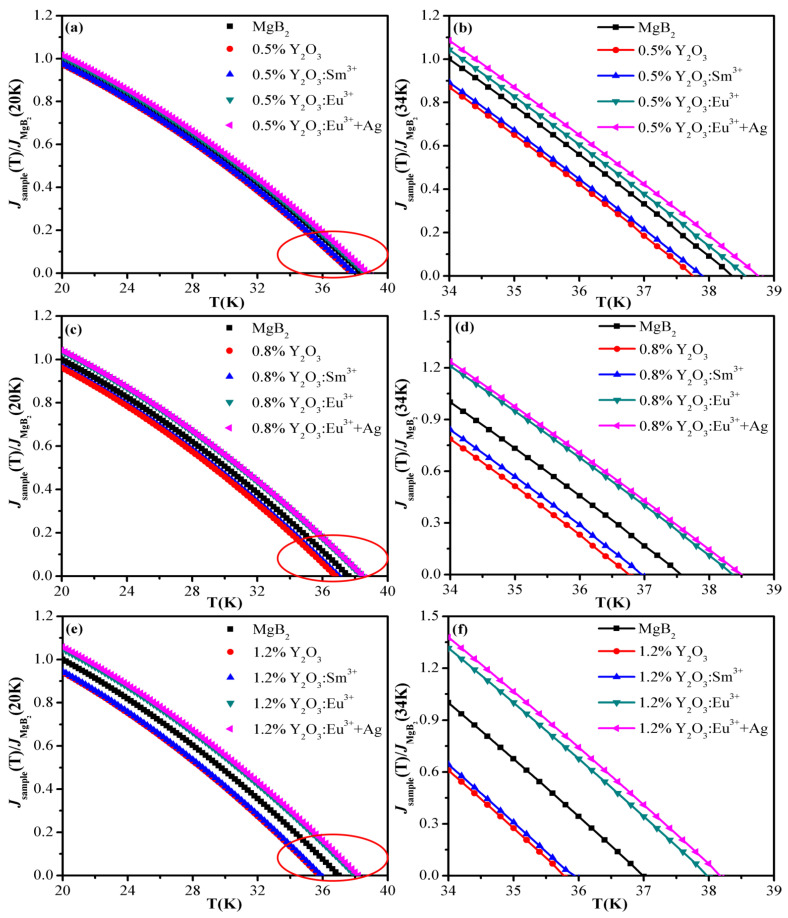
The relationship between *J_C_* and temperature of pure MgB_2_ and doping MgB_2_ samples. (**a**,**b**) The relationship between *J_C_* and temperature of pure ^a^MgB_2_ and ^a^MgB_2_ doped with 0.5 wt% Y_2_O_3_:Sm^3+^, Y_2_O_3_, Y_2_O_3_:Eu^3+^, and Y_2_O_3_:Eu^3+^+Ag. (**c**,**d**) The relationship between *J_C_* and temperature of pure ^b^MgB_2_ and ^b^MgB_2_ doped with 0.8 wt% Y_2_O_3_:Sm^3+^, Y_2_O_3_, Y_2_O_3_:Eu^3+^, and Y_2_O_3_:Eu^3+^+Ag. (**e**,**f**) The relationship between *J_C_* and temperature of pure ^c^MgB_2_ and ^c^MgB_2_ doped with 1.2 wt% Y_2_O_3_:Sm^3+^, Y_2_O_3_, Y_2_O_3_:Eu^3+^, and Y_2_O_3_:Eu^3+^+Ag. (**b**,**d**,**f**) are partial enlarged images.

Figure 2a,b show the relationship between *J_C_* and the temperature of pure ^a^MgB_2_ and ^a^MgB_2_ doped with 0.5 wt% Y_2_O_3_:Sm^3+^, Y_2_O_3_, Y_2_O_3_:Eu^3+^, and Y_2_O_3_:Eu^3+^+Ag. Figure 2c,d depict the relationship between *J_C_* and the temperature of pure ^b^MgB_2_ and ^b^MgB_2_ doped with 0.8 wt% Y_2_O_3_:Sm^3+^, Y_2_O_3_, Y_2_O_3_:Eu^3+^, and Y_2_O_3_:Eu^3+^+Ag. Figure 2e,f demonstrate the relationship between *J_C_* and the temperature of pure ^c^MgB_2_ and ^c^MgB_2_ doped with 1.2 wt% Y_2_O_3_:Sm^3+^, Y_2_O_3_, Y_2_O_3_:Eu^3+^, and Y_2_O_3_:Eu^3+^+Ag. The *J_C_* of ^a^MgB_2_, ^b^MgB_2_, and ^c^MgB_2_ are 8.9 × 10^4^, 7.8 × 10^4^, and 7.2 × 10^4^ A/cm^2^ at 20 K. As observed, the *J_C_* of pure MgB_2_ and doped samples decreases with the increase in temperature, which is consistent with the results of [28,47,48]. The *J_C_* of pure MgB_2_ is comparable to references [49,50]. The *J_C_* decreases slowly when the temperature is lower, and the decreasing rate increases with the rise in temperature. The doping of Y_2_O_3_:Eu^3+^ and Y_2_O_3_:Eu^3+^+Ag luminescent inhomogeneous phases increases the *J_C_*. The ^c^MgB_2_ series samples with the smallest particle size have a higher dopant content, and the most increases in *J_C_*. At *T* = 34 K, the *J_C_* of Y_2_O_3_:Eu^3+^ and Y_2_O_3_:Eu^3+^+Ag doped samples increases by 32% and 38% compared with purely that of ^c^MgB_2_, respectively. The *J_C_* of non-luminescent dopant-doped MgB_2_ samples first reduces to a minimum value, while the luminescent inhomogeneous phase-doped samples can have the *J_C_* at a higher temperature. For example, the *J_C_* of pure ^c^MgB_2_ reduces to a minimum value at 36.8 K, and the *J_C_* of 1.2 wt% Y_2_O_3_:Sm^3+^ and Y_2_O_3_ doped samples reduces to a minimum value at 35.8 and 35.6 K, while the *J_C_* of 1.2 wt% Y_2_O_3_:Eu^3+^ and Y_2_O_3_:Eu^3+^+Ag doped samples decreases to a minimum value at 37.8 and 38 K, respectively.

**Figure 3:**
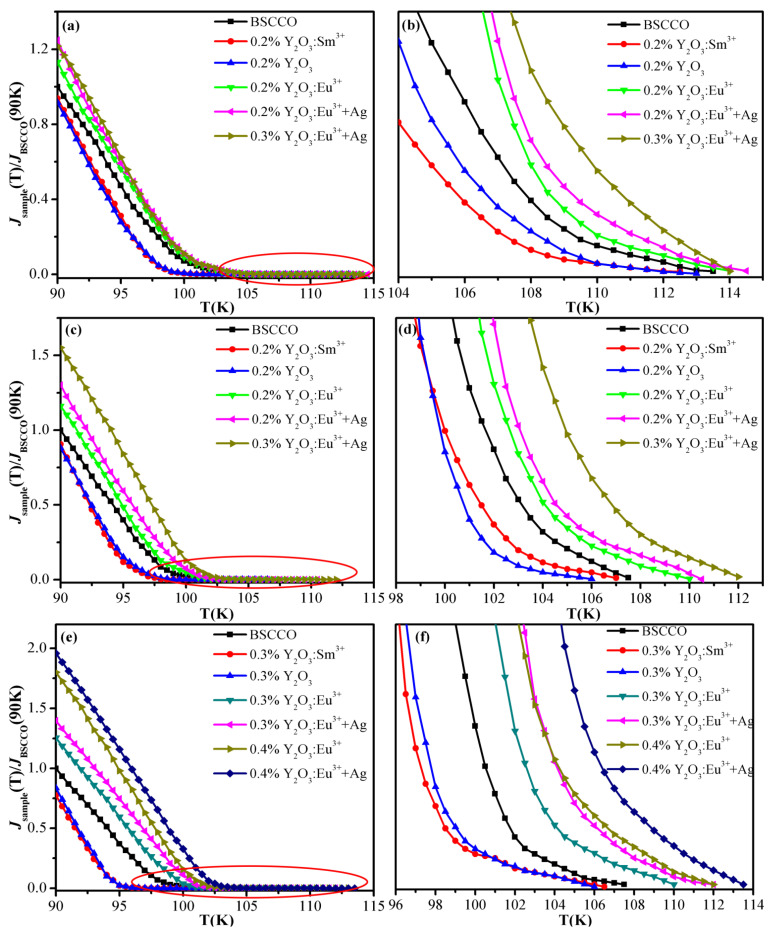
The relationship between *J_C_* and temperature of pure B(P)SCCO and doping B(P)SCCO samples. (**a**,**b**) The relationship between *J_C_* and temperature of pure B(P)SCCO (A1) and B(P)SCCO doped with 0.2 wt% Y_2_O_3_:Sm^3+^ (A2), Y_2_O_3_ (A3), Y_2_O_3_:Eu^3+^ (A4), Y_2_O_3_:Eu^3+^+Ag (A5), and 0.3 wt% Y_2_O_3_:Eu^3+^+Ag (A6). (**c**,**d**) The relationship between *J_C_* and temperature of pure B(P)SCCO (B1) and 0.2 wt% Y_2_O_3_:Sm^3+^ (B2), Y_2_O_3_ (B3), Y_2_O_3_:Eu^3+^ (B4), Y_2_O_3_:Eu^3+^+Ag (B5), and 0.3 wt% Y_2_O_3_:Eu^3+^+Ag (B6) doped samples. (**e**,**f**) The relationship between *J_C_* and temperature of pure B(P)SCCO (C1) and B(P)SCCO doped with 0.3 wt% Y_2_O_3_:Sm^3+^ (C2), Y_2_O_3_ (C3), Y_2_O_3_:Eu^3+^ (C4), Y_2_O_3_:Eu^3+^+Ag (C5), 0.4 wt% Y_2_O_3_:Eu^3+^ (C6), and 0.4 wt% Y_2_O_3_:Eu^3+^+Ag (C7). (**b**,**d**,**f**) are partial enlarged images.

Figure 3a,b represent the relationship between *J_C_* and temperature of pure B(P)SCCO (A1) and B(P)SCCO doped with 0.2 wt% Y_2_O_3_:Sm^3+^ (A2), Y_2_O_3_ (A3), Y_2_O_3_:Eu^3+^ (A4), Y_2_O_3_:Eu^3+^+Ag (A5), and 0.3 wt% Y_2_O_3_:Eu^3+^+Ag (A6). Figure 3c,d depict the relationship between *J_C_* and temperature of pure B(P)SCCO (B1) and 0.2 wt% Y_2_O_3_:Sm^3+^ (B2), Y_2_O_3_ (B3), Y_2_O_3_:Eu^3+^ (B4), Y_2_O_3_:Eu^3+^+Ag (B5), and 0.3 wt% Y_2_O_3_:Eu^3+^+Ag (B6) doped samples. Figure 3e,f demonstrate the relationship between *J_C_* and temperature of pure B(P)SCCO (C1) and B(P)SCCO doped with 0.3 wt% Y_2_O_3_:Sm^3+^ (C2), 0.3 wt% Y_2_O_3_ (C3), 0.3 wt% Y_2_O_3_:Eu^3+^ (C4), 0.3 wt% Y_2_O_3_:Eu^3+^+Ag (C5), 0.4 wt% Y_2_O_3_:Eu^3+^ (C6), and 0.4 wt% Y_2_O_3_:Eu^3+^+Ag (C7). The *J_C_* of B(P)SCCO (A1), B(P)SCCO (B1), and B(P)SCCO (C1) are 103, 70, and 54 A/cm^2^ at 90 K. The figures show that the *J_C_* of all samples decreases with the increase in temperature, which is consistent with [51,52]. The *J_C_* of all samples decreases rapidly at lower temperature and slows down at higher temperature. This finding is completely contrary to the results observed for the conventional superconductor MgB_2_. The *J_C_* of pure B(P)SCCO is comparable to that of references [52,53,54]. Y_2_O_3_:Eu^3+^ and Y_2_O_3_:Eu^3+^+Ag luminescent inhomogeneous phase doping increases the *J_C_* of B(P)SCCO, and the *J_C_* of C-series samples with the smallest particle size increases the most. At *T* = 90 K, the *J_C_* of Y_2_O_3_:Eu^3+^ and Y_2_O_3_:Eu^3+^+Ag luminescent inhomogeneous phase-doped samples increases by 80% and 95% compared with that of pure B(P)SCCO. Moreover, the *J_C_* of luminescent inhomogeneous phase-doped samples decreases to a minimum value at higher temperature, while the *J_C_* of non-luminescent dopant-doped samples decreases to a minimum value at lower temperature. For example, for C-series samples, the *J_C_* of pure B(P)SCCO (C1) reduces to a minimum value at 107.5 K, and the *J_C_* of 0.3 wt% Y_2_O_3_:Sm^3+^ (C2), Y_2_O_3_ (C3) doped samples reduces to a minimum value at 106.5 and 106 K, respectively. While the *J_C_* of 0.3 wt% Y_2_O_3_:Eu^3+^ (C4), 0.3 wt% Y_2_O_3_:Eu^3+^+Ag (C5), 0.4 wt% Y_2_O_3_:Eu^3+^ (C6), and 0.4 wt% Y_2_O_3_:Eu^3+^+Ag (C7) doped samples decreases to a minimum value at 110, 112, 112, and 113.5 K, respectively.

**Figure 4:**
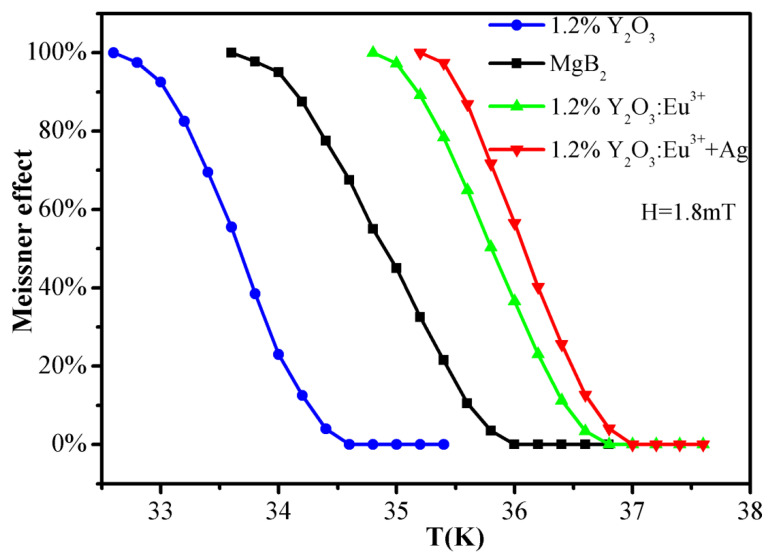
DC magnetization data of pure ^c^MgB_2_ and ^c^MgB_2_ doped with 1.2 wt% Y_2_O_3_, Y_2_O_3_:Eu^3+^, and Y_2_O_3_:Eu^3+^+Ag.

Figure 4 depicts the DC magnetization data of pure ^c^MgB_2_ and ^c^MgB_2_ doped with 1.2 wt% Y_2_O_3_, Y_2_O_3_:Eu^3+^, and Y_2_O_3_:Eu^3+^+Ag. Magnetization measurement shows that prepared samples have diamagnetism at a lower temperature. The diamagnetism of the superconductor can be represented by a Meissner effect, which is usually described in the literature by the relationship between the Meissner effect and temperature [46,55,56]. Therefore, we also showed the relationship between the Meissner effect and the temperature, as shown in Figure 4. The *Y*-axis is the percentage of the Meissner effect, indicating the strength of the Meissner effect (that is, the strength of the diamagnetism of the sample), and the *X*-axis is the temperature. The Meissner effect weakens and eventually disappears with the increase in temperature. The Meissner effect disappears at 36 K for pure ^c^MgB_2_ sample, and it disappears at 34.6 K for the ^c^MgB_2_ doped with non-luminescent dopant Y_2_O_3_. Meanwhile, the Meissner effect of Y_2_O_3_:Eu^3+^ and Y_2_O_3_:Eu^3+^+Ag luminescent inhomogeneous phase-doped ^c^MgB_2_ samples disappears when temperature is higher than 36.8 and 37 K, respectively.

**Figure 5:**
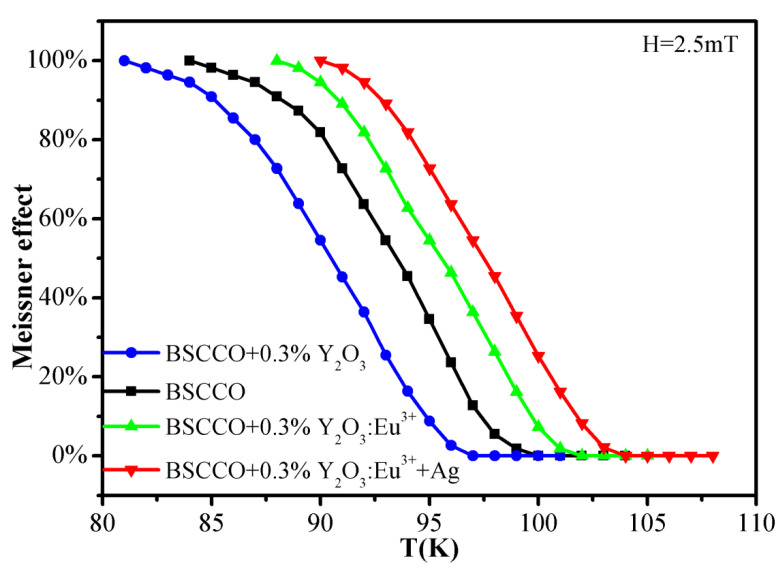
DC magnetization data of pure B(P)SCCO (C1) and B(P)SCCO doped with 0.3 wt% Y_2_O_3_ (C3), Y_2_O_3_:Eu^3+^ (C4), and Y_2_O_3_:Eu^3+^+Ag (C5).

Figure 5 shows the DC magnetization data of pure B(P)SCCO (C1) and B(P)SCCO doped with 0.3 wt% Y_2_O_3_ (C3), Y_2_O_3_:Eu^3+^ (C4), and Y_2_O_3_:Eu^3+^+Ag (C5). Meissner effect is observed in all Bi(Pb)SrCaCuO samples by DC magnetization data, and the Meissner effect weakens and eventually disappears with the increase in temperature, which is consistent with the results of references [57,58,59]. The Meissner effect of pure B(P)SCCO disappears when the temperature is higher than 100 K, and that of B(P)SCCO doped with non-luminescent dopant Y_2_O_3_ disappears when the temperature is higher than 97 K. Meanwhile, the Meissner effect of Y_2_O_3_:Eu^3+^ and Y_2_O_3_:Eu^3+^+Ag luminescent inhomogeneous phase-doped samples disappears when the temperature is higher than 102 and 104 K, respectively.

In conventional MgB_2_ and high-temperature copper oxide Bi(Pb)SrCaCuO superconductors, the sample doped with non-luminescent dopants has a Meissner effect at lower temperatures, while the Meissner effect is found in samples doped with a luminescent inhomogeneous phase at a higher temperature. Therefore, the superconductivity is enhanced by the doping of the luminescent inhomogeneous phase.

## 4. Conclusions

In this study, the *I*–*V* curves of Bi(Pb)SrCaCuO and MgB_2_ smart meta-superconductor are measured by a four-probe method, the transport critical current density *J_C_* is obtained and the changes in *J_C_* are explored, the Meissner effect is also studied by DC magnetization measurement; the conclusions are as follows:

1. Y_2_O_3_:Eu^3+^+Ag luminescent inhomogeneous phase doping increases the *J_C_* of ^c^MgB_2_ by 38% (*T* = 34 K), while the *J_C_* of non-luminescent dopant-doped samples decreases. The *J_C_* of pure ^c^MgB_2_ decreases to a minimum value at 36.8 K, and the *J_C_* of Y_2_O_3_:Eu^3+^ and Y_2_O_3_:Eu^3+^+Ag-doped samples decreases to a minimum value at 37.8 and 38 K, respectively. Meanwhile, the *J_C_* of Y_2_O_3_:Sm^3+^ and Y_2_O_3_-doped samples reduces to a minimum value at 35.8 and 35.6 K. The Meissner effect disappears at 36 K for pure ^c^MgB_2_ sample, and it disappears at 36.8 and 37 K for Y_2_O_3_:Eu^3+^ and Y_2_O_3_:Eu^3+^+Ag luminescent inhomogeneous phase-doped samples. Meanwhile, the Meissner effect disappears at 34.6 K for Y_2_O_3_ non-luminescent dopant-doped sample.
2. Y_2_O_3_:Eu^3+^+Ag luminescent inhomogeneous phase doping increases the *J_C_* of Bi(Pb)SrCaCuO by 95% (*T* = 90 K), the *J_C_* of non-luminescent dopants doped samples decreases. The *J_C_* of pure Bi(Pb)SrCaCuO (C1) decreases to a minimum value at 107.5 K. The *J_C_* of Y_2_O_3_:Eu^3+^ and Y_2_O_3_:Eu^3+^+Ag-doped samples decreases to a minimum value at 112 and 113.5 K, respectively. Meanwhile, the *J_C_* of Y_2_O_3_:Sm^3+^ and Y_2_O_3_-doped samples reduces to a minimum value at 106.5 and 106 K. The Meissner effect of pure B(P)SCCO (C1) disappears when the temperature is higher than 100 K. The Meissner effect of Y_2_O_3_:Eu^3+^ and Y_2_O_3_:Eu^3+^+Ag luminescent inhomogeneous phase-doped samples disappears when the temperature is higher than 102 and 104 K, while that of the Y_2_O_3_-doped sample disappears when the temperature is higher than 97 K.
3. The *T_C_* and *J_C_* of smart meta-superconductor MgB_2_ and Bi(Pb)SrCaCuO increase simultaneously. The *J_C_* of luminescent inhomogeneous phase-doped samples decreases to a minimum value at a higher temperature. A smart meta-superconductor has the Meissner effect at higher temperatures. All these findings indicate that the improvement in superconducting performance through a smart meta-superconductor is applicable to conventional and copper oxide high-temperature superconductors.

## Data Availability

The data presented in this study are available on request from the corresponding author.
